# Crystal structure and Hirshfeld surface analysis of 2-{[7-acetyl-4-cyano-6-hy­droxy-8-(4-meth­oxyphen­yl)-1,6-dimethyl-5,6,7,8-tetra­hydro­isoquinolin-3-yl­]sulfan­yl}acetic acid ethyl ester

**DOI:** 10.1107/S2056989022000378

**Published:** 2022-01-25

**Authors:** Elham A. Al-Taifi, Islam S. Marae, Yasser A. El-Ossaily, Shaaban K. Mohamed, Joel T. Mague, Mehmet Akkurt, Etify A. Bakhite

**Affiliations:** a Chemistry Department, Faculty of Science, Sana’a University, Sana’a, Yemen; b Chemistry Department, Faculty of Science, Assiut University, 71516 Assiut, Egypt; c Chemistry Department, College of Science, Jouf University, PO Box 2014.Sakaka, Saudi Arabia; d Chemistry and Environmental Division, Manchester Metropolitan University, Manchester, M1 5GD, England; e Chemistry Department, Faculty of Science, Minia University, 61519 El-Minia, Egypt; f Department of Chemistry, Tulane University, New Orleans, LA 70118, USA; g Department of Physics, Faculty of Sciences, Erciyes University, 38039 Kayseri, Turkey

**Keywords:** crystal structure, tetra­hydro­iso­quinoline, ethyl ester, hydrogen bond, C—H⋯π(ring)

## Abstract

In the title mol­ecule, C_25_H_28_N_2_O_5_S, (alternative name ethyl 2-{[7-acetyl-4-cyano-6-hy­droxy-8-(4-meth­oxy­phen­yl)-1,6-dimethyl-5,6,7,8-tetra­hydro­isoquinolin-3-yl]sulfanyl}­acetate) the 4-meth­oxy­phenyl group is disposed on one side of the bicyclic core and the oxygen atoms of the hydroxyl and acetyl groups are disposed on the other side. In the crystal, a layered structure parallel to the *ac* plane is generated by O—H⋯O and C—H⋯O hydrogen bonds plus C—H⋯π(ring) inter­actions.

## Abstract 2

The 4-meth­oxy­phenyl group is disposed on one side of the bicyclic core and the oxygen atoms of the hydroxyl and acetyl groups are disposed on the other. The unsaturated portion of the core adopts an envelope conformation. In the crystal, O—H⋯O and C—H⋯O hydrogen bonds form chains extending along the *a*-axis direction. These are linked into layers parallel to the *ac* plane by additional C—H⋯O hydrogen bonds and C—H⋯π(ring) inter­actions.

## Chemical context

Some tetra­hydro­iso­quinoline (THISQ) based compounds are of medicinal and biological importance, being used as anti­tumoral (Pingaew *et al.*, 2014 ▸; Castillo *et al.*, 2018 ▸), anti­fungal (Scott *et al.*, 2002 ▸) and anti-inflammatory agents (Siegfried *et al.*, 1989 ▸). Other tetra­hydro­iso­quinolines were used as inhibitors including B-raf^V600E^ or p38 kinase inhibitors (Lu *et al.*, 2016 ▸; Rosales *et al.*, 2007 ▸). The THISQ core can easily be functionalized to build other heterocyclic rings on the carbocyclic ring (Xu *et al.*, 2002 ▸; Carroll *et al.*, 2007 ▸; Demers *et al.*, 2008 ▸, Marae *et al.*, 2021*a* ▸). Recently, we have used some compounds related to THISQ as durable fluorescent dyes for cotton (Marae *et al.*, 2021*b* ▸). The widespread importance of these compounds motivated us to further study the THISQ core. Here we report the synthesis and crystal structure determination of the title compound. 
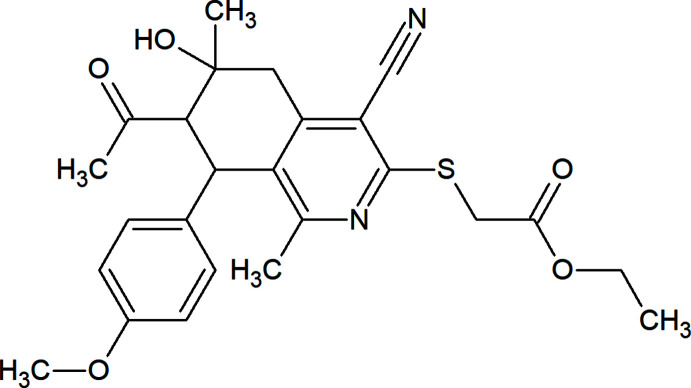


## Structural commentary

**Figure 1:**
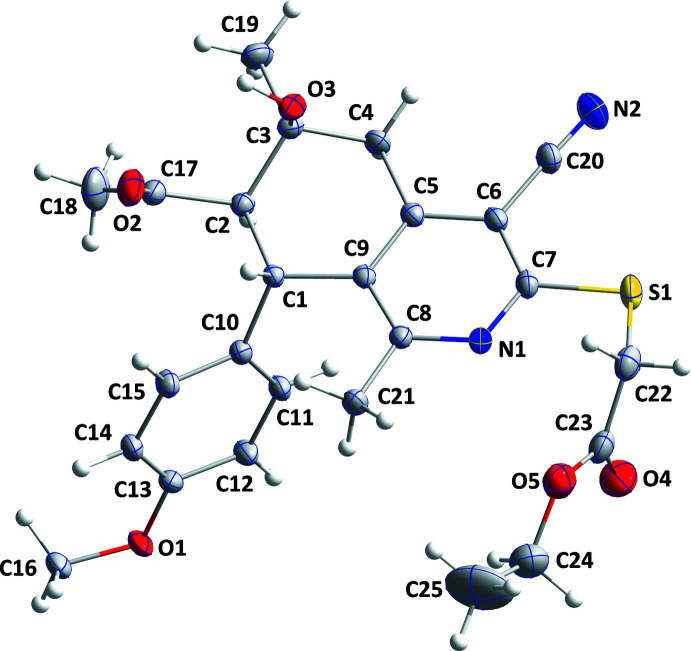
The title mol­ecule with labelling scheme and 50% probability ellipsoids.

The ethyl sulfanyl­acetate, acetyl and cyano groups and both methyl groups (C19 and C21) are in equatorial positions with respect to the bicyclic core, while the hydroxyl and anisole groups on the cyclo­hexane ring occupy an axial and bis­ectional position, respectively (Fig. 1 ▸). The C10–C15 benzene ring is inclined to the N1/C5–C9 pyridine ring by 82.57 (6)°. The C1–C5/C9 cyclo­hexane ring is in an envelope conformation, with atom C3 at the flap position [deviation from best plane = 0.367 (1) Å] and puckering parameters (Cremer & Pople, 1975 ▸) *Q*_T_ = 0.5180 (12) Å, θ = 53.85 (13)° and φ = 109.07 (17)°.

## Supra­molecular features

**Figure 2:**
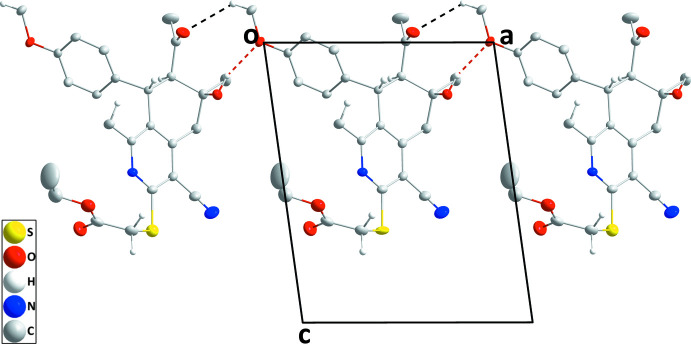
A portion of one chain viewed along the *b*-axis direction. O—H⋯O and C—H⋯O hydrogen bonds are depicted by red and black dashed lines, respectively.

**Figure 3:**
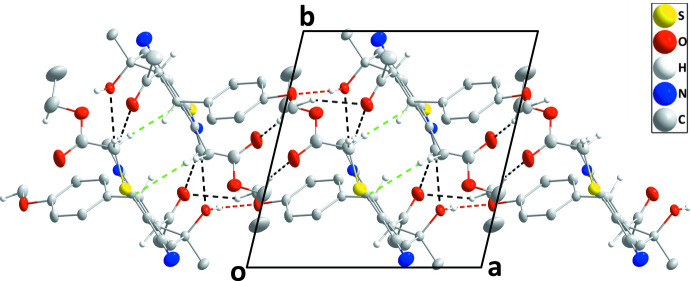
Packing viewed along the *c*-axis direction giving an elevation view of one layer. Hydrogen bonds are depicted as in Fig. 2 ▸ while C—H⋯π(ring) inter­actions are indicated by green dashed lines.

In the crystal of the title compound, chains of mol­ecules extending along the *a*-axis direction are formed by O3—H3⋯O1 and C16—H16*C*⋯O2 hydrogen bonds (Table 1 ▸ and Fig. 2 ▸). These are connected into layers parallel to the *ac* plane by C21—H21*A*⋯O2, C22—H22*A*⋯O3 and C24—H24*B*⋯O4 hydrogen bonds as well as C22—H22*B*⋯*Cg*1 inter­actions (Table 1 ▸ and Fig. 3 ▸).

## Hirshfeld surface analysis

**Figure 4:**
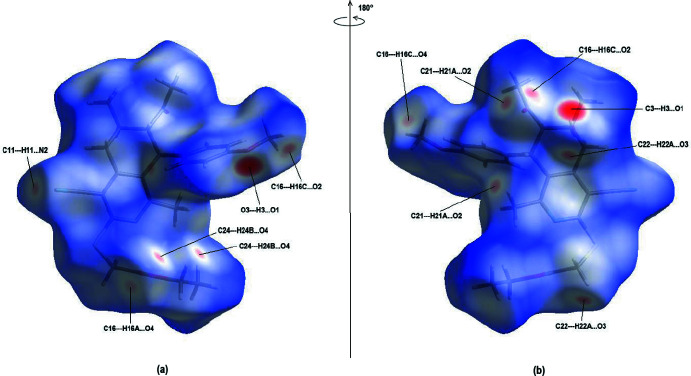
(*a*) Front and (*b*) back sides of the three-dimensional Hirshfeld surface of the title compound mapped over *d*_norm_, with a fixed colour scale of −0.4903 (red) to +1.6396 (blue) a.u.

Hirshfeld surface analysis (Spackman & Jayatilaka, 2009 ▸) was carried out using *CrystalExplorer17.5* (Turner *et al.*, 2017 ▸). The Hirshfeld surface and their associated two-dimensional fingerprint plots were used to qu­antify the various inter­molecular inter­actions in the title compound. In the Hirshfeld surface plotted over *d*_norm_ in the range −0.4903 (red) to +1.6396 (blue) a.u. (Fig. 4 ▸), the white areas indicate contacts with distances equal to the sum of van der Waals radii, and the red and blue areas indicate distances shorter (in close contact) or longer (distinct contact) than the van der Waals radii, respectively (Venkatesan *et al.*, 2016 ▸). The bright-red spots indicate their roles as the respective donors and/or acceptors.

**Figure 5:**
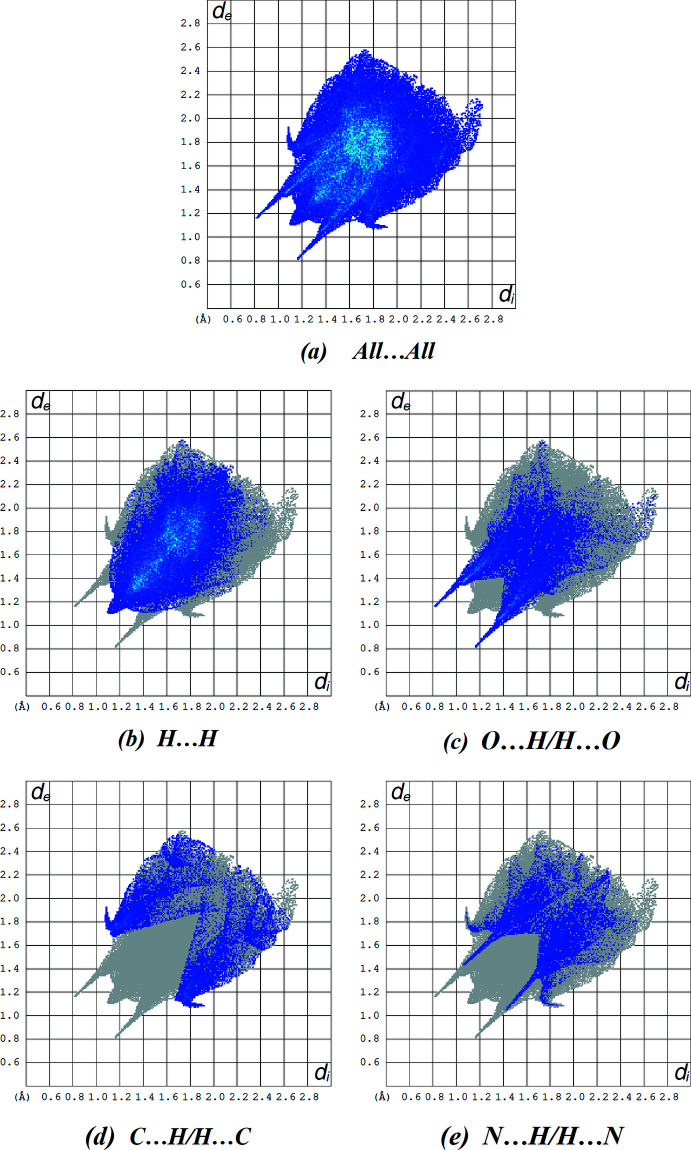
Two-dimensional fingerprint plots for the title compound, showing (*a*) all inter­actions, and delineated into (*b*) H⋯H, (*c*) O⋯H/H⋯O, (*d*) C⋯H/H⋯C and (*e*) N⋯H/H⋯N inter­actions. The *d*_i_ and *d*_e_ values are the closest inter­nal and external distances (in Å) from given points on the Hirshfeld surface.

Fingerprint plots (Fig. 5 ▸ *b*–*e*; Table 2 ▸) reveal that H⋯H (47.6%), O⋯H/H⋯O (19.7%), C⋯H/H⋯C (12.5%) and N⋯H/H⋯N (11.6%) inter­actions make the greatest contributions to the surface contacts. S⋯H/H⋯S (6.4%), N⋯C/C⋯N (0.7%), O⋯C/C⋯O (0.5%), O⋯O (0.5%) and C⋯C (0.4%) contacts also contribute to the overall crystal packing of the title compound. The Hirshfeld surface analysis confirms the importance of H-atom contacts in establishing the packing. The large number of H⋯H, O⋯H, C⋯H and N⋯H inter­actions suggest that van der Waals inter­actions and hydrogen bonding play the major roles in the crystal packing (Hathwar *et al.*, 2015 ▸).

## Database survey

A search of the Cambridge Structural Database (CSD version 5.42, updated September 2021; Groom *et al.*, 2016 ▸) for tetra­hydro­iso­quinoline derivatives gave nine compounds very similar to the title compound. In the crystal of NAQRIJ (Mague *et al.*, 2017 ▸), dimers form through complementary sets of inversion-related O—H⋯O and C—H⋯O hydrogen bonds. These are connected into zigzag chains along the *c*-axis direction by pairwise C—H⋯N inter­actions that also form inversion dimers. In KUGLIK (Langenohl *et al.*, 2020 ▸), the heterocyclic amines are alternately connected to the hydrogen-bonding system along the *c* axis, which leads to the formation of syndiotactic polymer chains in this direction. In the crystal of DUSVIZ (Selvaraj *et al.*, 2020 ▸), mol­ecules are linked *via* C—H⋯O hydrogen bonds. In AKIVUO (Al-Taifi *et al.*, 2021 ▸), a layered structure with layers parallel to (10 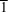 ) is generated by O—H⋯O and C—H⋯O hydrogen bonds. In ULUTAZ (Naghiyev *et al.*, 2021 ▸), mol­ecules are linked *via* N—H⋯O and C—H⋯N hydrogen bonds, forming a three-dimensional network, and the crystal packing is dominated by C—H⋯π bonds. In CARCOQ (Lehmann *et al.*, 2017 ▸), mol­ecules are linked by O—H⋯O hydrogen bonds, forming chains propagating along the *a*-axis direction. The chains are linked by C—H⋯F hydrogen bonds, forming layers lying parallel to the *ab* plane. In POPYEB (Ben Ali *et al.*, 2019 ▸), mol­ecules are packed in a herringbone manner parallel to (103) and (10 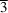 ) *via* weak C—H⋯O and C—H⋯π(ring) inter­actions. In ENOCIU (Naicker *et al.*, 2011 ▸) various C—H⋯π and C—H⋯O bonds link the mol­ecules together. In NIWPAL (Bouasla *et al.*, 2008 ▸), the mol­ecules are linked by N—H⋯O inter­molecular hydrogen bonds involving the sulfonamide function to form an infinite two-dimensional network parallel to the (001) plane.

## Synthesis and crystallization

7-Acetyl-4-cyano-1,6-dimethyl-6-hy­droxy-8-(4-meth­oxy­phen­yl)-5,6,7,8-tetra­hydro-iso­quinoline-3(2*H*)-thione (5 mmol, 1.91 g) and sodium acetate trihydrate (1.36 g, 10 mmol) were suspended in 50 ml of absolute ethanol, then 0.55 ml of ethyl chloro­acetate (5.3 mmol) were added and the mixture was refluxed for one h. During reflux, the yellow colour disappeared gradually over time to afford a colourless reaction mixture. The reaction mixture was then left to cool at room temperature and the formed precipitate was collected by fiitration, washed with water, dried in air and recystallized from ethanol to give the title compound as cubic crystals, yield 2.11 g (94%); m.p. 453–455 K. IR (cm^−1^): 3454 (O—H); 3048 (C—H aromatic); 2970, 2913 (C—H aliphatic); 2215 (C≡N); 1743 (C=O, ester); 1697 (C=O, acet­yl). ^1^H NMR (CDCl_3_, 400 MHz) δ: 6.80–6.86 (*dd*, *J* = 8 Hz, 4H, ArH), 4.24–4.26 (*d*, *J* = 8 Hz, 1H, C^8^H), 4.12–4.15 (*q*, *J* = 6 Hz, 2H, OCH_2_), 3.89–3.92 (*dd*, 2H, SCH_2_), 3.78 (s, 3H, OCH_3_), 3.38 (*s*, 1H, OH), 3.09–3.12 (*d*, *J* = 12 Hz, 1H, C^5^H), 3.03–3.05 (*d*, *J* = 8 Hz, 1H, C^7^H), 2.89–2.92 (*d*, *J* = 12 Hz, 1H, C^5^H), 1. 90 (*s*, 3H, CH_3_ at C-1), 1.80 (*s*, 3H, COCH_3_), 1.34 (*s*, 3H, CH_3_ at C-6), 1.18–1.21 (*t*, *J* = 6 Hz, 3H, CH_3_ of ester group).

## Refinement

Crystal data, data collection and structure refinement details are summarized in Table 3 ▸. All C-bound H atoms were placed in geometrically idealized positions (C—H = 0.95–1.00 Å) while the hydrogen atom attached to O3 was found from a difference map, and was subsequently refined isotropically [O3—H3 = 0.903 (17) Å] with *U*_iso_(H) = 1.5*U*_eq_(O). All C-bound H atoms were included as riding contributions with isotropic displacement parameters 1.2 times those of the parent atoms (1.5 for methyl groups).

## Supplementary Material

Crystal structure: contains datablock(s) I, global. DOI: 10.1107/S2056989022000378/vm2259sup1.cif

Structure factors: contains datablock(s) I. DOI: 10.1107/S2056989022000378/vm2259Isup2.hkl

Supporting information file. DOI: 10.1107/S2056989022000378/vm2259Isup3.cml

CCDC reference: 2141278

Additional supporting information:  crystallographic information; 3D view; checkCIF report

## Figures and Tables

**Table 1:** Hydrogen-bond geometry (Å, °) *Cg*1 is the centroid of the N1/C5–C9 pyridine ring.

*D*—H⋯*A*	*D*—H	H⋯*A*	*D*⋯*A*	*D*—H⋯*A*
O3—H3⋯O1^i^	0.90 (2)	2.05 (2)	2.9283 (12)	164 (2)
C16—H16*C*⋯O2^ii^	0.98	2.47	3.1566 (15)	127
C21—H21*A*⋯O2^iii^	0.98	2.51	3.3956 (15)	150
C22—H22*A*⋯O3^iv^	0.99	2.44	3.1815 (15)	131
C22—H22*B*⋯*Cg*1^iv^	0.99	2.58	3.4559 (15)	147
C24—H24*B*⋯O4^v^	0.99	2.52	3.442 (2)	154

Symmetry codes: (i) x+1, y, z; (ii) x-1, y, z; (iii) -x+1, -y+1, -z; (iv) -x+1, -y+1, -z+1; (v) -x, -y+1, -z+1.

**Table 2:** Summary of short inter­atomic contacts (Å) in the title compound

Contact	Distance	Symmetry operation
O1⋯H3	2.051 (16)	−1 + *x*, *y*, *z*
H21*A*⋯O2	2.51	1 − *x*, 1 − *y*, −*z*
H22*A*⋯O3	2.44	1 − *x*, 1 − *y*, 1 − *z*
O4⋯H16*A*	2.60	*x*, *y*, 1 + *z*
H24*B*⋯H24*B*	2.44	−*x*, 1 − *y*, 1 − *z*
H11⋯N2	2.61	1 − *x*, − *y*, 1 − *z*
H18*B*⋯H2	2.49	1 − *x*, − *y*, −*z*
H21*C*⋯H16*B*	2.51	−*x*, 1 − *y*, −*z*
H25*B*⋯H25*B*	2.34	−*x*, 2 − *y*, 1 − *z*

**Table 3:** Experimental details

Crystal data	
Chemical formula	C_25_H_28_N_2_O_5_S
*M*_r_	468.55
Crystal system, space group	Triclinic, *P*\overline{1}
Temperature (K)	150
*a*, *b*, *c* (Å)	10.0643 (6), 10.3592 (7), 12.0685 (8)
α, β, γ (°)	83.296 (1), 80.770 (1), 75.638 (1)
*V* (Å^3^)	1199.23 (13)
*Z*	2
Radiation type	Mo *K*α
μ (mm^−1^)	0.17
Crystal size (mm)	0.35 × 0.29 × 0.27

Data collection	
Diffractometer	Bruker SMART APEX CCD
Absorption correction	Multi-scan (*SADABS*; Krause *et al.*, 2015 ▸)
*T*_min_, *T*_max_	0.82, 0.96
No. of measured, independent and observed [*I* > 2σ(*I*)] reflections	22695, 6509, 5177
*R*_int_	0.023
(sin θ/λ)_max_ (Å^−1^)	0.695

Refinement	
*R*[*F* ^2^ > 2σ(*F* ^2^)], *wR*(*F* ^2^), *S*	0.045, 0.133, 1.11
No. of reflections	6509
No. of parameters	305
H-atom treatment	H atoms treated by a mixture of independent and constrained refinement
Δρ_max_, Δρ_min_ (e Å^−3^)	0.71, −0.22

Computer programs: *APEX3* and *SAINT* (Bruker, 2016 ▸), *SHELXT2014/5* (Sheldrick, 2015*a* ▸), *SHELXL2018/3* (Sheldrick, 2015*b* ▸), *DIAMOND* (Brandenburg & Putz, 2012 ▸) and *SHELXTL* (Sheldrick, 2008 ▸).

## supplementary crystallographic information

### Crystal data

**Table:**

C_25_H_28_N_2_O_5_S	*Z* = 2
*M**_r_* = 468.55	*F*(000) = 496
Triclinic, *P*1	*D*_x_ = 1.298 Mg m^−^^3^
*a* = 10.0643 (6) Å	Mo *K*α radiation, λ = 0.71073 Å
*b* = 10.3592 (7) Å	Cell parameters from 9995 reflections
*c* = 12.0685 (8) Å	θ = 2.5–29.5°
α = 83.296 (1)°	µ = 0.17 mm^−^^1^
β = 80.770 (1)°	*T* = 150 K
γ = 75.638 (1)°	Block, colourless
*V* = 1199.23 (13) Å^3^	0.35 × 0.29 × 0.27 mm

### Data collection

**Table:**

Bruker SMART APEX CCD diffractometer	6509 independent reflections
Radiation source: fine-focus sealed tube	5177 reflections with *I* > 2σ(*I*)
Graphite monochromator	*R*_int_ = 0.023
Detector resolution: 8.3333 pixels mm^-1^	θ_max_ = 29.6°, θ_min_ = 1.7°
φ and ω scans	*h* = −13→13
Absorption correction: multi-scan (*SADABS*; Krause *et al.*, 2015)	*k* = −14→14
*T*_min_ = 0.82, *T*_max_ = 0.96	*l* = −16→16
22695 measured reflections	

### Refinement

**Table:**

Refinement on *F*^2^	Primary atom site location: dual
Least-squares matrix: full	Secondary atom site location: difference Fourier map
*R*[*F*^2^ > 2σ(*F*^2^)] = 0.045	Hydrogen site location: inferred from neighbouring sites
*wR*(*F*^2^) = 0.133	H atoms treated by a mixture of independent and constrained refinement
*S* = 1.11	*w* = 1/[σ^2^(*F*_o_^2^) + (0.0848*P*)^2^ + 0.0389*P*] where *P* = (*F*_o_^2^ + 2*F*_c_^2^)/3
6509 reflections	(Δ/σ)_max_ < 0.001
305 parameters	Δρ_max_ = 0.71 e Å^−^^3^
0 restraints	Δρ_min_ = −0.22 e Å^−^^3^

### Special details

**Table:**

Experimental. The diffraction data were obtained from 3 sets of 400 frames, each of width 0.5° in ω, colllected at φ = 0.00, 90.00 and 180.00° and 2 sets of 800 frames, each of width 0.45° in φ, collected at ω = –30.00 and 210.00°. The scan time was 10 sec/frame.
Geometry. All esds (except the esd in the dihedral angle between two l.s. planes) are estimated using the full covariance matrix. The cell esds are taken into account individually in the estimation of esds in distances, angles and torsion angles; correlations between esds in cell parameters are only used when they are defined by crystal symmetry. An approximate (isotropic) treatment of cell esds is used for estimating esds involving l.s. planes.
Refinement. Refinement of F^2^ against ALL reflections. The weighted R-factor wR and goodness of fit S are based on F^2^, conventional R-factors R are based on F, with F set to zero for negative F^2^. The threshold expression of F^2^ > 2sigma(F^2^) is used only for calculating R-factors(gt) etc. and is not relevant to the choice of reflections for refinement. R-factors based on F^2^ are statistically about twice as large as those based on F, and R- factors based on ALL data will be even larger. H-atoms attached to carbon were placed in calculated positions (C—H = 0.95 - 1.00 Å) while that attached to oxygen was placed in a location derived from a difference map and its coordinates adjusted to give O—H = 0.87 %A. All were included as riding contributions with isotropic displacement parameters 1.2 - 1.5 times those of the attached atoms.

### Fractional atomic coordinates and isotropic or equivalent isotropic displacement parameters (Å^2^)

**Table:**

	*x*	*y*	*z*	*U*_iso_*/*U*_eq_	
S1	0.40179 (4)	0.33194 (3)	0.67126 (2)	0.02919 (11)	
O1	−0.01601 (8)	0.26808 (9)	−0.00282 (7)	0.0261 (2)	
O2	0.65454 (10)	0.31103 (10)	−0.03628 (7)	0.0325 (2)	
O3	0.76674 (8)	0.24179 (8)	0.18545 (7)	0.02199 (18)	
H3	0.8202 (16)	0.2524 (10)	0.1190 (13)	0.033*	
O4	0.08897 (11)	0.46076 (12)	0.66388 (9)	0.0455 (3)	
O5	0.13749 (11)	0.65264 (10)	0.58156 (8)	0.0357 (2)	
N1	0.35085 (10)	0.41231 (10)	0.46279 (8)	0.0208 (2)	
N2	0.67128 (14)	0.03469 (14)	0.60676 (11)	0.0424 (3)	
C1	0.46954 (11)	0.29768 (11)	0.16884 (9)	0.0159 (2)	
H1	0.500590	0.379658	0.134272	0.019*	
C2	0.57706 (11)	0.17513 (11)	0.12120 (9)	0.0173 (2)	
H2	0.531350	0.098468	0.129582	0.021*	
C3	0.70498 (11)	0.13262 (11)	0.18391 (9)	0.0193 (2)	
C4	0.65493 (12)	0.09343 (12)	0.30675 (10)	0.0223 (2)	
H4A	0.620266	0.011333	0.310543	0.027*	
H4B	0.734138	0.072924	0.350225	0.027*	
C5	0.54209 (11)	0.20148 (11)	0.35998 (9)	0.0179 (2)	
C6	0.52467 (11)	0.20985 (12)	0.47729 (9)	0.0199 (2)	
C7	0.42631 (12)	0.31658 (12)	0.52466 (9)	0.0205 (2)	
C8	0.36570 (11)	0.40524 (11)	0.35083 (9)	0.0177 (2)	
C9	0.45724 (11)	0.29873 (11)	0.29599 (9)	0.0166 (2)	
C10	0.33305 (11)	0.30117 (11)	0.12715 (9)	0.0176 (2)	
C11	0.24375 (12)	0.22431 (12)	0.18378 (9)	0.0206 (2)	
H11	0.263234	0.176107	0.253526	0.025*	
C12	0.12671 (12)	0.21705 (12)	0.13990 (10)	0.0227 (2)	
H12	0.065702	0.165748	0.180301	0.027*	
C13	0.09882 (11)	0.28515 (12)	0.03646 (9)	0.0200 (2)	
C14	0.18526 (12)	0.36397 (12)	−0.02017 (9)	0.0225 (2)	
H14	0.165733	0.412138	−0.089914	0.027*	
C15	0.30109 (12)	0.37198 (12)	0.02605 (9)	0.0212 (2)	
H15	0.359409	0.427055	−0.012431	0.025*	
C16	−0.03346 (13)	0.32073 (14)	−0.11635 (10)	0.0267 (3)	
H16A	0.052176	0.288044	−0.166573	0.040*	
H16B	−0.054905	0.418620	−0.120457	0.040*	
H16C	−0.109566	0.291484	−0.139534	0.040*	
C17	0.61828 (12)	0.20825 (12)	−0.00420 (10)	0.0227 (2)	
C18	0.60948 (18)	0.11357 (16)	−0.08583 (12)	0.0404 (4)	
H18A	0.654485	0.138974	−0.160555	0.061*	
H18B	0.512060	0.117187	−0.089559	0.061*	
H18C	0.656149	0.022519	−0.060849	0.061*	
C19	0.81182 (13)	0.01430 (13)	0.13312 (11)	0.0280 (3)	
H19A	0.849094	0.041750	0.056329	0.042*	
H19B	0.767456	−0.059489	0.131300	0.042*	
H19C	0.887278	−0.015108	0.179227	0.042*	
C20	0.60828 (13)	0.11221 (13)	0.54794 (10)	0.0258 (3)	
C21	0.27853 (12)	0.52205 (12)	0.29046 (10)	0.0236 (2)	
H21A	0.327912	0.540938	0.215809	0.035*	
H21B	0.259623	0.600477	0.333793	0.035*	
H21C	0.190956	0.501257	0.282226	0.035*	
C22	0.31143 (14)	0.50489 (13)	0.67068 (10)	0.0286 (3)	
H22A	0.305095	0.534976	0.746654	0.034*	
H22B	0.366449	0.558138	0.617296	0.034*	
C23	0.16744 (14)	0.53363 (14)	0.63858 (10)	0.0303 (3)	
C24	0.00183 (17)	0.68891 (18)	0.54348 (14)	0.0490 (4)	
H24A	−0.072036	0.696185	0.608938	0.059*	
H24B	−0.007797	0.619864	0.497063	0.059*	
C25	−0.0099 (3)	0.8197 (2)	0.4757 (3)	0.0934 (9)	
H25A	0.062343	0.810824	0.410224	0.140*	
H25B	0.001445	0.886882	0.522048	0.140*	
H25C	−0.101112	0.847711	0.450117	0.140*	

### Atomic displacement parameters (Å^2^)

**Table:**

	*U*^11^	*U*^22^	*U*^33^	*U*^12^	*U*^13^	*U*^23^
S1	0.0375 (2)	0.03615 (19)	0.01391 (15)	−0.00874 (14)	−0.00410 (13)	−0.00088 (12)
O1	0.0185 (4)	0.0418 (5)	0.0196 (4)	−0.0100 (4)	−0.0083 (3)	0.0052 (4)
O2	0.0360 (5)	0.0411 (6)	0.0226 (5)	−0.0181 (4)	0.0007 (4)	0.0018 (4)
O3	0.0192 (4)	0.0271 (4)	0.0219 (4)	−0.0090 (3)	−0.0012 (3)	−0.0056 (3)
O4	0.0337 (6)	0.0612 (7)	0.0426 (6)	−0.0229 (5)	−0.0002 (5)	0.0106 (5)
O5	0.0392 (5)	0.0333 (5)	0.0325 (5)	−0.0047 (4)	−0.0021 (4)	−0.0057 (4)
N1	0.0213 (5)	0.0254 (5)	0.0155 (4)	−0.0052 (4)	−0.0023 (4)	−0.0012 (4)
N2	0.0461 (7)	0.0444 (7)	0.0312 (6)	−0.0021 (6)	−0.0128 (6)	0.0117 (5)
C1	0.0158 (5)	0.0182 (5)	0.0142 (5)	−0.0049 (4)	−0.0033 (4)	0.0001 (4)
C2	0.0163 (5)	0.0195 (5)	0.0168 (5)	−0.0050 (4)	−0.0017 (4)	−0.0027 (4)
C3	0.0171 (5)	0.0204 (5)	0.0206 (5)	−0.0041 (4)	−0.0022 (4)	−0.0033 (4)
C4	0.0207 (5)	0.0217 (6)	0.0214 (6)	−0.0006 (4)	−0.0038 (4)	0.0020 (4)
C5	0.0174 (5)	0.0193 (5)	0.0177 (5)	−0.0061 (4)	−0.0037 (4)	0.0013 (4)
C6	0.0188 (5)	0.0237 (6)	0.0171 (5)	−0.0059 (4)	−0.0049 (4)	0.0034 (4)
C7	0.0230 (5)	0.0254 (6)	0.0145 (5)	−0.0091 (5)	−0.0029 (4)	0.0008 (4)
C8	0.0158 (5)	0.0223 (5)	0.0154 (5)	−0.0053 (4)	−0.0023 (4)	−0.0012 (4)
C9	0.0154 (5)	0.0203 (5)	0.0153 (5)	−0.0064 (4)	−0.0030 (4)	−0.0002 (4)
C10	0.0168 (5)	0.0206 (5)	0.0151 (5)	−0.0031 (4)	−0.0032 (4)	−0.0018 (4)
C11	0.0194 (5)	0.0267 (6)	0.0156 (5)	−0.0060 (4)	−0.0050 (4)	0.0033 (4)
C12	0.0183 (5)	0.0314 (6)	0.0193 (5)	−0.0099 (5)	−0.0038 (4)	0.0048 (5)
C13	0.0152 (5)	0.0271 (6)	0.0175 (5)	−0.0030 (4)	−0.0040 (4)	−0.0020 (4)
C14	0.0216 (5)	0.0291 (6)	0.0157 (5)	−0.0045 (5)	−0.0054 (4)	0.0031 (4)
C15	0.0218 (5)	0.0250 (6)	0.0175 (5)	−0.0086 (4)	−0.0036 (4)	0.0034 (4)
C16	0.0222 (6)	0.0387 (7)	0.0197 (6)	−0.0058 (5)	−0.0095 (5)	0.0020 (5)
C17	0.0189 (5)	0.0308 (6)	0.0182 (5)	−0.0050 (5)	−0.0016 (4)	−0.0040 (5)
C18	0.0571 (10)	0.0423 (8)	0.0242 (7)	−0.0115 (7)	−0.0059 (6)	−0.0121 (6)
C19	0.0229 (6)	0.0268 (6)	0.0309 (7)	0.0021 (5)	−0.0023 (5)	−0.0085 (5)
C20	0.0283 (6)	0.0292 (6)	0.0191 (6)	−0.0073 (5)	−0.0042 (5)	0.0043 (5)
C21	0.0238 (6)	0.0243 (6)	0.0194 (5)	0.0011 (5)	−0.0040 (5)	−0.0011 (4)
C22	0.0335 (7)	0.0344 (7)	0.0209 (6)	−0.0127 (5)	−0.0005 (5)	−0.0081 (5)
C23	0.0315 (7)	0.0387 (7)	0.0197 (6)	−0.0099 (6)	0.0045 (5)	−0.0059 (5)
C24	0.0383 (8)	0.0555 (10)	0.0449 (9)	0.0022 (7)	−0.0026 (7)	−0.0031 (8)
C25	0.0751 (16)	0.0498 (12)	0.139 (3)	0.0112 (11)	−0.0281 (16)	0.0228 (14)

### Geometric parameters (Å, º)

**Table:**

S1—C7	1.7672 (11)	C10—C11	1.3927 (15)
S1—C22	1.7966 (14)	C11—C12	1.3882 (16)
O1—C13	1.3742 (14)	C11—H11	0.9500
O1—C16	1.4355 (14)	C12—C13	1.3940 (15)
O2—C17	1.2116 (15)	C12—H12	0.9500
O3—C3	1.4223 (14)	C13—C14	1.3863 (16)
O3—H3	0.903 (17)	C14—C15	1.3944 (16)
O4—C23	1.2046 (17)	C14—H14	0.9500
O5—C23	1.3298 (17)	C15—H15	0.9500
O5—C24	1.457 (2)	C16—H16A	0.9800
N1—C7	1.3240 (15)	C16—H16B	0.9800
N1—C8	1.3439 (14)	C16—H16C	0.9800
N2—C20	1.1443 (17)	C17—C18	1.4956 (18)
C1—C9	1.5206 (14)	C18—H18A	0.9800
C1—C10	1.5278 (15)	C18—H18B	0.9800
C1—C2	1.5501 (15)	C18—H18C	0.9800
C1—H1	1.0000	C19—H19A	0.9800
C2—C17	1.5258 (15)	C19—H19B	0.9800
C2—C3	1.5421 (15)	C19—H19C	0.9800
C2—H2	1.0000	C21—H21A	0.9800
C3—C4	1.5290 (16)	C21—H21B	0.9800
C3—C19	1.5311 (15)	C21—H21C	0.9800
C4—C5	1.5028 (16)	C22—C23	1.5093 (19)
C4—H4A	0.9900	C22—H22A	0.9900
C4—H4B	0.9900	C22—H22B	0.9900
C5—C9	1.3941 (15)	C24—C25	1.488 (3)
C5—C6	1.4087 (15)	C24—H24A	0.9900
C6—C7	1.3972 (16)	C24—H24B	0.9900
C6—C20	1.4369 (16)	C25—H25A	0.9800
C8—C9	1.4053 (15)	C25—H25B	0.9800
C8—C21	1.4957 (15)	C25—H25C	0.9800
C10—C15	1.3893 (15)		
			
C7—S1—C22	98.39 (6)	C13—C14—C15	119.48 (10)
C13—O1—C16	116.26 (9)	C13—C14—H14	120.3
C3—O3—H3	109.5	C15—C14—H14	120.3
C23—O5—C24	115.10 (12)	C10—C15—C14	121.43 (11)
C7—N1—C8	119.27 (10)	C10—C15—H15	119.3
C9—C1—C10	113.57 (9)	C14—C15—H15	119.3
C9—C1—C2	113.46 (9)	O1—C16—H16A	109.5
C10—C1—C2	106.92 (8)	O1—C16—H16B	109.5
C9—C1—H1	107.5	H16A—C16—H16B	109.5
C10—C1—H1	107.5	O1—C16—H16C	109.5
C2—C1—H1	107.5	H16A—C16—H16C	109.5
C17—C2—C3	111.24 (9)	H16B—C16—H16C	109.5
C17—C2—C1	108.37 (9)	O2—C17—C18	121.16 (12)
C3—C2—C1	112.73 (9)	O2—C17—C2	120.04 (11)
C17—C2—H2	108.1	C18—C17—C2	118.78 (11)
C3—C2—H2	108.1	C17—C18—H18A	109.5
C1—C2—H2	108.1	C17—C18—H18B	109.5
O3—C3—C4	106.22 (9)	H18A—C18—H18B	109.5
O3—C3—C19	110.37 (9)	C17—C18—H18C	109.5
C4—C3—C19	109.61 (10)	H18A—C18—H18C	109.5
O3—C3—C2	111.05 (9)	H18B—C18—H18C	109.5
C4—C3—C2	107.54 (9)	C3—C19—H19A	109.5
C19—C3—C2	111.84 (9)	C3—C19—H19B	109.5
C5—C4—C3	112.68 (9)	H19A—C19—H19B	109.5
C5—C4—H4A	109.1	C3—C19—H19C	109.5
C3—C4—H4A	109.1	H19A—C19—H19C	109.5
C5—C4—H4B	109.1	H19B—C19—H19C	109.5
C3—C4—H4B	109.1	N2—C20—C6	177.83 (14)
H4A—C4—H4B	107.8	C8—C21—H21A	109.5
C9—C5—C6	118.33 (10)	C8—C21—H21B	109.5
C9—C5—C4	121.92 (10)	H21A—C21—H21B	109.5
C6—C5—C4	119.67 (10)	C8—C21—H21C	109.5
C7—C6—C5	119.09 (10)	H21A—C21—H21C	109.5
C7—C6—C20	119.89 (10)	H21B—C21—H21C	109.5
C5—C6—C20	121.00 (11)	C23—C22—S1	114.39 (9)
N1—C7—C6	122.29 (10)	C23—C22—H22A	108.7
N1—C7—S1	116.98 (9)	S1—C22—H22A	108.7
C6—C7—S1	120.69 (9)	C23—C22—H22B	108.7
N1—C8—C9	122.66 (10)	S1—C22—H22B	108.7
N1—C8—C21	113.87 (10)	H22A—C22—H22B	107.6
C9—C8—C21	123.45 (10)	O4—C23—O5	124.65 (13)
C5—C9—C8	118.17 (10)	O4—C23—C22	124.79 (13)
C5—C9—C1	121.80 (10)	O5—C23—C22	110.53 (11)
C8—C9—C1	119.86 (9)	O5—C24—C25	107.60 (17)
C15—C10—C11	118.27 (10)	O5—C24—H24A	110.2
C15—C10—C1	120.46 (10)	C25—C24—H24A	110.2
C11—C10—C1	121.02 (9)	O5—C24—H24B	110.2
C12—C11—C10	121.02 (10)	C25—C24—H24B	110.2
C12—C11—H11	119.5	H24A—C24—H24B	108.5
C10—C11—H11	119.5	C24—C25—H25A	109.5
C11—C12—C13	119.91 (10)	C24—C25—H25B	109.5
C11—C12—H12	120.0	H25A—C25—H25B	109.5
C13—C12—H12	120.0	C24—C25—H25C	109.5
O1—C13—C14	124.12 (10)	H25A—C25—H25C	109.5
O1—C13—C12	116.04 (10)	H25B—C25—H25C	109.5
C14—C13—C12	119.84 (10)		
			
C9—C1—C2—C17	−159.86 (9)	C21—C8—C9—C5	174.35 (10)
C10—C1—C2—C17	74.15 (10)	N1—C8—C9—C1	−179.57 (10)
C9—C1—C2—C3	−36.30 (12)	C21—C8—C9—C1	−0.96 (16)
C10—C1—C2—C3	−162.29 (9)	C10—C1—C9—C5	125.98 (11)
C17—C2—C3—O3	67.55 (12)	C2—C1—C9—C5	3.61 (14)
C1—C2—C3—O3	−54.40 (12)	C10—C1—C9—C8	−58.88 (13)
C17—C2—C3—C4	−176.63 (9)	C2—C1—C9—C8	178.74 (9)
C1—C2—C3—C4	61.42 (12)	C9—C1—C10—C15	143.67 (11)
C17—C2—C3—C19	−56.24 (13)	C2—C1—C10—C15	−90.40 (12)
C1—C2—C3—C19	−178.19 (9)	C9—C1—C10—C11	−42.15 (14)
O3—C3—C4—C5	64.88 (12)	C2—C1—C10—C11	83.77 (12)
C19—C3—C4—C5	−175.88 (10)	C15—C10—C11—C12	0.81 (17)
C2—C3—C4—C5	−54.09 (12)	C1—C10—C11—C12	−173.49 (10)
C3—C4—C5—C9	23.77 (15)	C10—C11—C12—C13	1.36 (18)
C3—C4—C5—C6	−153.08 (10)	C16—O1—C13—C14	9.11 (16)
C9—C5—C6—C7	−1.73 (16)	C16—O1—C13—C12	−170.76 (10)
C4—C5—C6—C7	175.24 (10)	C11—C12—C13—O1	177.43 (10)
C9—C5—C6—C20	179.59 (11)	C11—C12—C13—C14	−2.44 (18)
C4—C5—C6—C20	−3.44 (17)	O1—C13—C14—C15	−178.52 (11)
C8—N1—C7—C6	2.08 (18)	C12—C13—C14—C15	1.34 (18)
C8—N1—C7—S1	179.98 (8)	C11—C10—C15—C14	−1.94 (17)
C5—C6—C7—N1	−1.67 (18)	C1—C10—C15—C14	172.40 (10)
C20—C6—C7—N1	177.03 (11)	C13—C14—C15—C10	0.87 (18)
C5—C6—C7—S1	−179.50 (8)	C3—C2—C17—O2	−73.61 (14)
C20—C6—C7—S1	−0.80 (16)	C1—C2—C17—O2	50.84 (14)
C22—S1—C7—N1	−15.41 (11)	C3—C2—C17—C18	107.90 (13)
C22—S1—C7—C6	162.54 (10)	C1—C2—C17—C18	−127.65 (12)
C7—N1—C8—C9	0.94 (17)	C7—S1—C22—C23	69.08 (10)
C7—N1—C8—C21	−177.79 (10)	C24—O5—C23—O4	−3.71 (19)
C6—C5—C9—C8	4.47 (16)	C24—O5—C23—C22	178.31 (11)
C4—C5—C9—C8	−172.42 (10)	S1—C22—C23—O4	36.21 (17)
C6—C5—C9—C1	179.69 (10)	S1—C22—C23—O5	−145.80 (9)
C4—C5—C9—C1	2.79 (16)	C23—O5—C24—C25	−176.43 (17)
N1—C8—C9—C5	−4.26 (16)		

### Hydrogen-bond geometry (Å, º)

*Cg*1 is the centroid of the N1/C5–C9 pyridine ring.

**Table:**

*D*—H···*A*	*D*—H	H···*A*	*D*···*A*	*D*—H···*A*
O3—H3···O1^i^	0.90 (2)	2.05 (2)	2.9283 (12)	164 (2)
C16—H16*C*···O2^ii^	0.98	2.47	3.1566 (15)	127
C21—H21*A*···O2^iii^	0.98	2.51	3.3956 (15)	150
C22—H22*A*···O3^iv^	0.99	2.44	3.1815 (15)	131
C22—H22*B*···*Cg*1^iv^	0.99	2.58	3.4559 (15)	147
C24—H24*B*···O4^v^	0.99	2.52	3.442 (2)	154

Symmetry codes: (i) *x*+1, *y*, *z*; (ii) *x*−1, *y*, *z*; (iii) −*x*+1, −*y*+1, −*z*; (iv) −*x*+1, −*y*+1, −*z*+1; (v) −*x*, −*y*+1, −*z*+1.

## References

[bb1] Al-Taifi, E. A., Maraei, I. S., Bakhite, E. A., Demirtas, G., Mague, J. T., Mohamed, S. K. & Ramli, Y. (2021). *Acta Cryst.* E**77**, 121–125. 10.1107/S2056989021000372 PMC7869536 33614138

[bb2] Ben Ali, K. & Retailleau, P. (2019). *Acta Cryst.* E**75**, 1399–1402. 10.1107/S2056989019011964 PMC6775741 31636965

[bb3] Bouasla, R., Berredjem, M., Aouf, N.-E. & Barbey, C. (2008). *Acta Cryst.* E**64**, o432. 10.1107/S1600536807068158 PMC2960235 21201459

[bb4] Brandenburg, K. & Putz, H. (2012). *DIAMOND*, Crystal Impact GbR, Bonn, Germany.

[bb5] Bruker (2016). *APEX3*, *SADABS* and *SAINT*. Bruker AXS Inc., Madison, Wisconsin, USA.

[bb6] Carroll, F. I., Robinson, T. P., Brieaddy, L. E., Atkinson, R. N., Mascarella, S. W., Damaj, M. I., Martin, B. R. & Navarro, H. A. (2007). *J. Med. Chem.* **50**, 6383–6391. 10.1021/jm0704696 17994682

[bb7] Castillo, J.-C., Jiménez, E., Portilla, J., Insuasty, B., Quiroga, J., Moreno-Fuquen, R., Kennedy, A. R. & Abonia, R. (2018). *Tetrahedron*, **74**, 932–947.

[bb8] Cremer, D. & Pople, J. A. (1975). *J. Am. Chem. Soc.* **97**, 1354–1358.

[bb9] Demers, S., Stevenson, H., Candler, J., Bashore, C. G., Arnold, E. P., O’Neill, B. T. & Coe, J. W. (2008). *Tetrahedron Lett.* **49**, 3368–3371.

[bb10] Groom, C. R., Bruno, I. J., Lightfoot, M. P. & Ward, S. C. (2016). *Acta Cryst.* B**72**, 171–179. 10.1107/S2052520616003954 PMC4822653 27048719

[bb11] Hathwar, V. R., Sist, M., Jørgensen, M. R. V., Mamakhel, A. H., Wang, X., Hoffmann, C. M., Sugimoto, K., Overgaard, J. & Iversen, B. B. (2015). *IUCrJ*, **2**, 563–574. 10.1107/S2052252515012130 PMC4547824 26306198

[bb12] Krause, L., Herbst-Irmer, R., Sheldrick, G. M. & Stalke, D. (2015). *J. Appl. Cryst.* **48**, 3–10. 10.1107/S1600576714022985 PMC4453166 26089746

[bb13] Langenohl, F., Otte, F. & Strohmann, C. (2020). *Acta Cryst.* E**76**, 298–302. 10.1107/S2056989020000730 PMC7057357 32148864

[bb14] Lehmann, A., Lechner, L., Radacki, K., Braunschweig, H. & Holzgrabe, U. (2017). *Acta Cryst.* E**73**, 867–870. 10.1107/S2056989017007186 PMC5458312 28638647

[bb15] Lu, B., Cao, H., Cao, J., Huang, S., Hu, Q., Liu, D., Shen, R., Shen, X., Tao, W., Wan, H., Wang, D., Yan, Y., Yang, L., Zhang, J., Zhang, L., Zhang, L. & Zhang, M. (2016). *Bioorg. Med. Chem. Lett.* **26**, 819–823. 10.1016/j.bmcl.2015.12.086 26739779

[bb16] Mague, J. T., Mohamed, S. K., Akkurt, M., Bakhite, E. A. & Albayati, M. R. (2017). *IUCrData*, **2**, x170390.

[bb17] Marae, I. S., Bakhite, E. A., Moustafa, O. S., Abbady, M. S., Mohamed, S. K. & Mague, J. T. (2021*a*). *ACS Omega*, **6**, 8706–8716. 10.1021/acsomega.1c00601 PMC8015098 33817534

[bb18] Marae, I. S., Sharmoukh, W., Bakhite, E. A., Moustafa, O. S., Abbady, M. S. & Emam, H. (2021*b*). *Cellulose*, **28**, 5937–5956.

[bb19] Naghiyev, F. N., Grishina, M. M., Khrustalev, V. N., Khalilov, A. N., Akkurt, M., Akobirshoeva, A. A. & Mamedov, İ. G. (2021). *Acta Cryst.* E**77**, 195–199. 10.1107/S2056989021000785 PMC7869549 33614153

[bb20] Naicker, T., Govender, T., Kruger, H. G. & Maguire, G. E. M. (2011). *Acta Cryst.* C**67**, o100–o103. 10.1107/S0108270110053357 21368406

[bb21] Pingaew, R., Mandi, P., Nantasenamat, C., Prachayasittikul, S., Ruchirawat, S. & Prachayasittikul, V. (2014). *Eur. J. Med. Chem.* **81**, 192–203. 10.1016/j.ejmech.2014.05.019 24836071

[bb22] Rosales, A. & Bernado, V. (2007). *Pyrazoloisoquinoline Derivatives*. WIPO Patent WO2007/060198A12007.

[bb23] Scott, J. D. & Williams, R. (2002). *Chem. Rev.* **102**, 1669–1730. 10.1021/cr010212u 11996547

[bb24] Selvaraj, J. P., Mary, S., Dhruba, J. B., Huidrom, B. S., Panneerselvam, Y. & Piskala Subburaman, K. (2020). *Acta Cryst.* E**76**, 1548–1550. 10.1107/S2056989020010300 PMC7534224 33117561

[bb25] Sheldrick, G. M. (2008). *Acta Cryst.* A**64**, 112–122. 10.1107/S0108767307043930 18156677

[bb26] Sheldrick, G. M. (2015*a*). *Acta Cryst.* A**71**, 3–8.

[bb27] Sheldrick, G. M. (2015*b*). *Acta Cryst.* C**71**, 3–8.

[bb28] Siegfried, L., Helmut, V., Guenther, W., Thomas, S., Eckart, S., Dieter, L., Gunter, L. & Ger East, D. D. (1989). *Chem. Abstr.* **110**, 75554g.

[bb29] Spackman, M. A. & Jayatilaka, D. (2009). *CrystEngComm*, **11**, 19–32.

[bb30] Turner, M. J., McKinnon, J. J., Wolff, S. K., Grimwood, D. J., Spackman, M. A., Jayatilaka, D. & Spackman, M. A. (2017). *Crystal Explorer*. University of Western Australia.

[bb31] Venkatesan, P., Thamotharan, S., Ilangovan, A., Liang, H. & Sundius, T. (2016). *Spectrochim. Acta A*, **153**, 625–636. 10.1016/j.saa.2015.09.002 26452098

[bb32] Xu, R., Dwoskin, L. P., Grinevich, V., Sumithran, S. P. & Crooks, P. A. (2002). *Drug Dev. Res.* **55**, 173–186.

